# The Development of Hyaluronan/Fucoidan-Based Nanoparticles as Macrophages Targeting an Epigallocatechin-3-Gallate Delivery System

**DOI:** 10.3390/ijms21176327

**Published:** 2020-08-31

**Authors:** Chang-Hsun Ho, Pei-Yi Chu, Shin-Lei Peng, Shun-Chih Huang, Yu-Hsin Lin

**Affiliations:** 1 Department of Anesthesiology, Show Chwan Memorial Hospital, Changhua 50008, Taiwan; jikeo2003@gmail.com; 2 Faculty of Pharmacy, National Yang-Ming University, Taipei 11221, Taiwan; ositachu@ym.edu.tw (P.-Y.C.); strawtarry@gmail.com (S.-C.H.); 3 Department of Biomedical Imaging and Radiological Science, China Medical University, Taichung 40402, Taiwan; speng@mail.cmu.edu.tw; 4 Department of Medical Research, China Medical University, Taichung 404332, Taiwan; 5 Department and Institute of Pharmacology, Center for Advanced Pharmaceutics and Drug Delivery Research, Institute of Biopharmaceutical Sciences, National Yang-Ming University, Taipei 11221, Taiwan

**Keywords:** macrophage-targeted nanoparticle, hyaluronan, fucoidan, epigallocatechin-3-gallate, CD44, anti-inflammatory

## Abstract

The aim of this study was to develop a macrophage-targeted nanoparticle composed of hyaluronan/fucoidan complexes with polyethylene glycol-gelatin to encapsulate and deliver epigallocatechin-3-gallate (EGCG), a compound that can regulate macrophage activation and pro-inflammatory mediator production. We show that our nanoparticles can successfully bond to macrophages and deliver more EGCG than an EGCG solution treatment, confirming the anti-inflammatory effects of these nanoparticles in lipopolysaccharide-stimulated macrophages. The prepared nanoparticles were established with a small mean particle size (217.00 ± 14.00 nm), an acceptable polydispersity index (0.28 ± 0.07), an acceptable zeta potential value (−33.60 ± 1.30 mV), and a high EGCG loading efficiency (52.08% ± 5.37%). The targeting abilities of CD44 binding were increased as the hyaluronan concentration increased and decreased by adding a competitor CD44 antibody. Moreover, we found that fucoidan treatment significantly reduced macrophage migration after lipopolysaccharide treatment in a dose-responsive manner. In summary, we successfully created macrophage-targeted nanoparticles for effective targeted delivery of EGCG, which should aid in the development of future anti-inflammatory drugs against macrophage-related diseases.

## 1. Introduction

Macrophages are a type of white blood cell of the immune system derived from monocytes that are located in nearly all tissues that specialize in clearing senescent or apoptotic cells, carrying out phagocytosis of pathogens, and maintaining homeostasis [1,2,3]. The balance of pro-inflammatory and anti-inflammatory programs is often the key to the outcomes of inflammatory diseases [4]. Accordingly, drugs and drug delivery strategies that target macrophages are gaining traction for the treatment inflammatory diseases. Nanoscale delivery systems, in particular, are promising platforms for macrophage-based immunomodulation due to their large surface areas, high drug-loading capacities, drug-stabilizing potential, prolonged circulation, and potentially enhanced accumulation and penetration in the diseased tissues [5]. Furthermore, modifying the delivery systems with ligands specific to the macrophage receptors, such as αvβ3 integrin, CD36, CD44, mannose receptors, class A scavenger receptors, and macrophage galactose type C-type lectin (MGL, CD301), has shown to improve the targeting efficacy [6,7]. Meanwhile, the immune system recognizes different pathogens with particular characteristics, such as peptidoglycan on gram-positive bacteria or lipopolysaccharides (LPS) on gram-negative bacteria. The initial host response to invading pathogenic bacteria includes acute neutrophil inflammatory influx, followed by the recruitment of macrophages [8]. LPS, a bacterial cell wall component, regulates CD44 expression and may modulate CD44-mediated biological effects in monocytic cells during inflammation and immune responses [9]. CD44 is also involved in several important physiological functions in cell-cell and cell-matrix interactions, including proliferation, adhesion, migration, and lymphocyte activation [10]. CD44 is a surface membrane glycoprotein that binds to hyaluronan, a component of the extracellular matrix, and the engagement of CD44 by immune cells with hyaluronan is a key event in inflammatory responses. Hyaluronan is composed of repeating (1→3)-β-D-GlcNAc and (1→4)-β-D-GlcA residues, and it has been proposed to specifically bind to CD44 receptors overexpressed on the surfaces of macrophages [8,11,12]. Fucoidans are sulfated polysaccharides present in seaweeds. They were first identified in brown algae in 1913 [13]. Fucoidan is commonly isolated from the brown seaweeds such as *Ascopbyllum nodosum*, *Fucus vesiculosus*, *Saccharina japonica*, and *Sargassum thunbergii* [14]. In fucoidans, the sulfate groups or uronic acids are bonded to _L_-fucose, with hundreds to thousands of _L_-fucose units linked by α-1-2 and α-1-4 bonds [15]. Fucoidan has numerous proven bioactivities, such as antioxidant, anticoagulant, antiviral, and anticancer activities [16]. These bioactivities are linked to the molecular weight, composition (e.g., monosaccharide composition, the degree of sulfation), and structure (glycosidic linkages, the degree of branching, and substitution, chain conformation, etc.) [17,18,19]. Xue et al. used fucoidan extracted from *Fucus vesiculosus* to inhibit mouse breast cancer cell growth in vivo and in vitro via the downregulation of the Wnt/β-catenin signaling pathway, without causing cytotoxic effects in the normal cells [20]. Studies have shown that fucoidan is a ligand for macrophage scavenger receptor A, which inhibits the production of nitric oxide (NO) when taken up by macrophages. Additional anti-inflammatory effects of fucoidan include the inhibition of leukocyte migration to inflammatory tissues and the modification of the migration activity of macrophages. Moreover, fucoidan can be extracted from marine organisms, including brown algae species, and has been marketed as a dietary supplement, or nutraceutical [21,22].

Among various medicinal and culinary plants, some species are of particular interest because of their ability to produce phytochemical-containing raw materials or preparations with significant antioxidant capacities and other health benefits [23,24]. Tea, rich in catechins, is known to act as an antioxidant, either through the chelation of metals with redox properties or as scavengers of free radicals [25]. The principal catechins in tea are (−)-epigallocatechin 3-gallate (EGCG), (−)-epicatechin 3-gallate (ECG), (−)-epigallocatechin (EGC), and (−)-epicatechin (EC). In addition to their antioxidant capabilities, tea catechins also have neuroprotective effects, can inhibit tumor angiogenesis, prevent atherosclerosis, and regulate cholesterol metabolism [26]. EGCG comprises 25 to 55% of green tea catechins and has been shown to have strong anti-inflammatory effects [27,28]. Studies exploring the mechanisms by which EGCG functions to attenuate inflammation have found that EGCG downregulates the phosphorylation of nuclear factor-kappa B (NF-κB), a key regulator of the classical pathway of the inflammation response induced by LPS [29]. However, poor bioavailability, susceptibility to oxidation, first-pass metabolism, and rapid efflux have been some of the major obstacles to its widespread use in drug development [30,31]. Therefore, an efficient delivery system is required to improve the targeting and bioavailability of EGCG. In light of this, key tasks for current EGCG research include finding appropriate target sites for the delivery and maintenance of an adequate cell fluid concentration.

As described in a previous study, we successfully produced EGCG-encapsulated nanoparticles [32]. We showed that these nanoparticles significantly enhanced EGCG stability, reduced the expression of cancer cell proliferation proteins, and controlled the release of EGCG to suppress tumor growth. In the current study, we developed a macrophage-targeting nanoparticle system of hyaluronan and fucoidan including polyethylene glycol-grafted gelatin (PEG-gelatin) to encapsulate the green tea polyphenol EGCG. Gelatin and PEG are water soluble and have biocompatible nontoxic properties. Gelatin has previously been used to bind polyphenols through hydrogen bonding interactions between hydrophobic proline residues and polyphenols on phenol rings [33]. Herein, we investigated the interplay mechanisms of hyaluronan and fucoidan molecules in binding CD44 proteins and inhibiting the migration of macrophage cells. Moreover, we examined the physicochemical characteristics of the prepared nanoparticles using Fourier transform infrared spectroscopy (FTIR) and dynamic light scattering. We showed that the nanoparticles could effectively carry EGCG and subsequently affect the expression of inflammation proteins, as determined by fluorescence microscopy and Western blot analysis.

## 2. Results

### 2.1. Effects of Fucoidan on Cell Migration Ability

**Figure 1:**
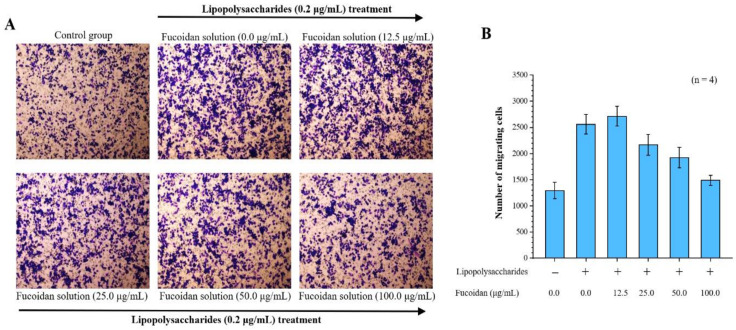
Anti-cell migration efficacy of fucoidan. Migrated cells were stained with 0.2% crystal violet. (**A**) Microscopic images of migrated RAW 264.7 cells at 24 h (4 replicates). Magnification: 100×; (**B**) Quantitative analysis of stained migrated cancer cells using Metamorph software are presented as means ± standard deviations (SD) (*n* = 4).

By investigating the effects of fucoidan on macrophage inflammation, we found that lipopolysaccharide significantly induced migration in macrophages. Our Transwell migration analysis revealed that 2560 ± 187 cells migrated to the lower surface after LPS treatment, compared with only 1294 ± 156 control cells. Notably, in the Transwell migration assay, there were 2715 ± 189, 2169 ± 201, 1924 ± 194, and 1489 ± 98 cells, corresponding to 12.5, 25.0, 50.0, and 100.0 μg/mL fucoidan treatment doses, respectively. Thus, fucoidan treatment significantly decreased macrophage migration in a dose-dependent manner (Figure 1).

### 2.2. Effects of Hyaluronan on CD44-Targeting Ability

**Figure 2:**
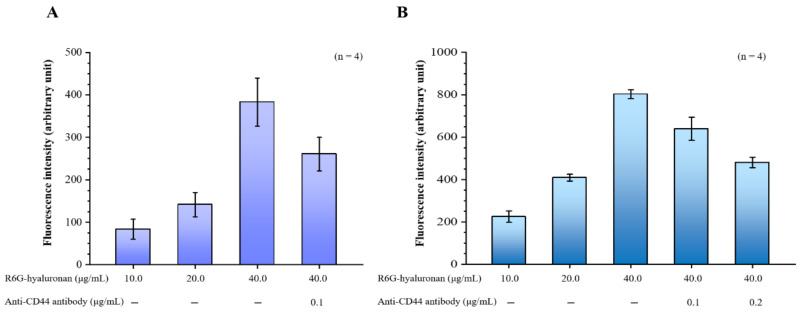
(**A**) Binding assays of fluorescence R6G-hyaluronan to immobilized recombinant CD44; (**B**) Binding assays of macrophages were incubated with fluorescence R6G-hyaluronan. Fluorescence intensities of Rh6G were detected. Data are expressed as means ± SDs (*n* = 4).

Our hyaluronan to CD44 binding assay revealed that the binding of hyaluronan to CD44 increased with the treatment dose of rhodamine 6G (R6G)-hyaluronan and significantly decreased by adding its competitor, the CD44 antibody. Furthermore, the binding of R6G-hyaluronan to macrophages was also examined (Figure 2A). The fluorescence intensity was 224.52 ± 26.86 to 803.11 ± 20.76 at a R6G-hyaluronan concentration of 10 to 40 μg/mL, indicating that the hyaluronan-receiving macrophage cells showed concentration dependence. Upon the addition of a competing CD44 protein, the adhesion of macrophages to hyaluronan was reduced by ~20–40% due to blockage by competition with the anti-CD44 antibody (Figure 2B). Therefore, we evaluated the benefits of the relative compositions of fucoidan and hyaluronan in macrophage-targeted nanoparticles.

### 2.3. Synthesis and Characterization of the EGCG-Loaded *Hyaluronan*/Fucoidan/PEG–Gelatin Nanoparticles

**Figure 3:**
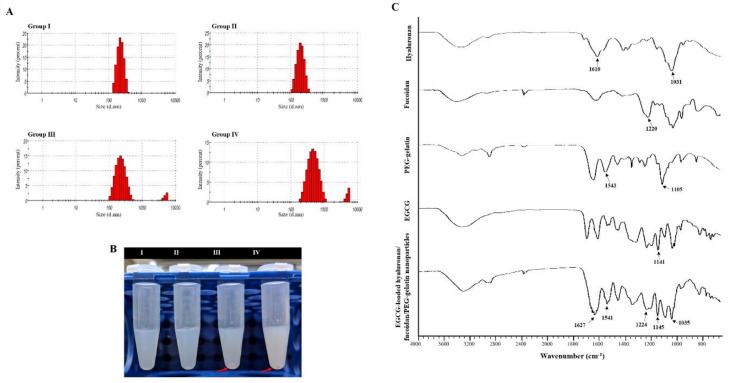
Characteristics of the prepared epigallocatechin-3-gallate (EGCG)-loaded hyaluronan/fucoidan/PEG–gelatin nanoparticles. (**A**) Particle size distributions; (**B**) Liquid morphology of EGCG-loaded hyaluronan/fucoidan/PEG–gelatin nanoparticles produced with different hyaluronan/fucoidan concentrations, as follows: Group I: 0.625:0.625:3.750:2.000; Group II: 1.250:1.250:3.750:2.000; Group III: 1.875:1.875:3.750:2.000; Group IV: 2.500:2.500:3.750:2.000 mg/mL; (**C**) Fourier transform infrared analyses of hyaluronan, fucoidan, PEG–gelatin, EGCG, and EGCG-loaded hyaluronan/fucoidan/PEG–gelatin nanoparticles.

To further determine the optimal composition of nanoparticles, formulations fabricated with varying proportions of hyaluronan and fucoidan by weight were examined. Table 1 shows how the weight ratio of fucoidan and hyaluronan impacted these parameters. The nanoparticle composed with hyaluronan/fucoidan/PEG–gelatin/EGCG proportions of 1.250:1.250:3.750:2.000 had a lower mean particle size (217.00 ± 14.00 nm), an acceptable zeta potential value (−33.60 ± 1.30 mV), and efficient EGCG loading (52.08 ± 5.37%) (Table 1). However, when the fucoidan/hyaluronan concentration was elevated to 1.875:1.875 and 2.500:2.500 mg/mL, some aggregates of the nanoparticle suspension were precipitated (red arrow) (Figure 3B). Subsequently, the particle size and polydispersity index of aqueous hyaluronan/fucoidan/PEG–gelatin/EGCG nanoparticles were 279.00 ± 16.00 nm and 0.38 ± 0.02 mV for 1.875:1.875:3.750:2.000 mg/mL, and 454.00 ± 28.00 nm and 0.45 ± 0.10 mV for 2.500:2.500:3.750:2.000 mg/mL, respectively, indicating a high level of heterogeneity (Table 1 and Figure 3A).

**Figure 4:**
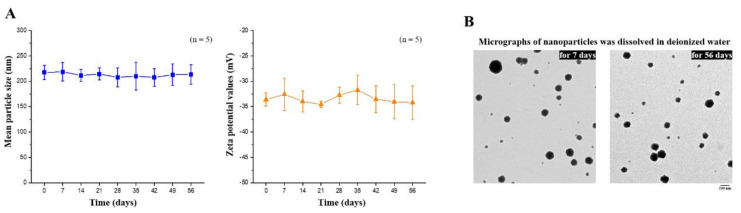
(**A**) Mean particle size and zeta potential values of EGCG-loaded nanoparticles were recorded for 56 days. Data are expressed as means ± SDs (*n* = 5); (**B**) Transmission electron microscopy micrographs of EGCG-loaded nanoparticles were observed after storage for 7 or 56 days. Scale bar: 200 nm.

The composition of the EGCG-loaded targeting nanoparticles was confirmed by FTIR analysis (Figure 3C). The spectra of hyaluronate, fucoidan, PEG–gelatin, and EGCG exhibited different peaks, representing the different characteristic stretches of bonds. The hyaluronate, by virtue of its carboxyl group, exhibited C=O asymmetric stretching vibration at 1610 cm^−1^ and C−O symmetric stretching vibration at 1031 cm^−1^. The spectrum of fucoidan presented a stretching vibration of sulfate esters at broad band O=S=O at 1220 cm^−1^. Symmetrical PEG–gelatin peaks at vibrations of C−O−C at 1105 cm^−1^ and −NH at 1543 cm^−1^ indicated the characteristic peaks of PEG and gelatin, respectively. The EGCG spectrum presented characteristic peaks of C−OH alcohol stretching vibrations at 1141 cm^−1^. The spectra of EGCG-loaded hyaluronan/fucoidan/PEG–gelatin nanoparticles revealed that prominent peaks at 1141 cm^−1^ shifted to 1145 cm^−1^, corresponding to C−OH deformation of EGCG, and the peak at 1543 cm^−1^ shifted to 1541 cm^−1^, which was attributed to the stretching vibration of the −NH bending on PEG–gelatin, reflecting that hydrogen bonds were formed by the bonding of N atoms of PEG–gelatin (C−OH···N−C) by interaction. Moreover, the characteristic C=O stretching of hyaluronate at 1610 cm^−1^ and the S=O stretching of fucoidan at 1220 cm^−1^ shifted to 1627 cm^−1^ and 1224 cm^−1^. These observations reflect intramolecular and intermolecular hydrogen bonding between –NH of PEG–gelatin to C=O of hyaluronate (C=O···H–N) and S=O of fucoidan (S=O···H–N). Furthermore, the mean particle size and zeta potential value of the prepared nanoparticles dissolved in water were examined every 7 days over a span of 56 days. These observations revealed that the conformation of hyaluronan/fucoidan/PEG–gelatin nanoparticles loaded with EGCG were stable for eight weeks, forming a spherical and uniform matrix (Figure 4).

### 2.4. Macrophage Cellular Internalization of Nanoparticles and Uptake of EGCG

**Figure 5:**
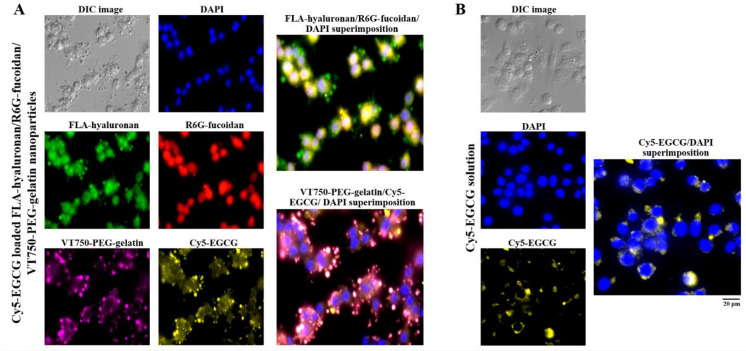
The intakes and distributions were observed and quantified using fluorescence microscopy. (**A**) Fluorescence nanoparticles composed of fluoresceinamine (FLA)-hyaluronan, R6G-fucoidan, VT750-PEG–gelatin, and Cy5-EGCG were given to macrophages and (**B**) Cy5-EGCG solution was given to macrophages. The cell nuclei were stained with 4′,6-diamidino-2-phenylindole (DAPI). Scale bar: 20 μm.

To observe the nanoparticle distribution and cellular uptake of EGCG in cells, macrophages were observed using fluorescence microscopy after treatment with cyanine 5 hydrazide (Cy5)-EGCG-loaded fluoresceinamine (FLA)-hyaluronan/R6G-fucoidan/VivoTag 750-NHS ester (VT750)-PEG–gelatin nanoparticles containing fluorescent materials (FLA (green), R6G (red), VT750 (purple), and Cy5 (yellow). A blue fluorescent stain, 4′,6-diamidino-2-phenylindole (DAPI), was used to label the cell nuclei. Figure 5A shows via fluorescence that FLA-hyaluronan and R6G–fucoidan were co-localized, had access to the cytoplasm, and slightly adhered to the nuclei. Moreover, the internalization of the Cy5-EGCG and VT750-PEG–gelatin fluorescence signals was observed in the intercellular space and cell cytoplasm, indicating that the gelatin–polyphenol complex was formed primarily via hydrogen bonding, which occurred because of the interaction between hydrophobic amino acids (mainly proline and residues) and the phenol rings of polyphenols. Furthermore, comparison of the delivery efficacy of Cy5-EGCG solution and Cy5-EGCG-loaded nanoparticles after macrophage cell treatment showed that there was more EGCG present in the macrophages in the EGCG-loaded nanoparticle treatment group than in the EGCG solution treatment group (Figure 5).

**Figure 6:**
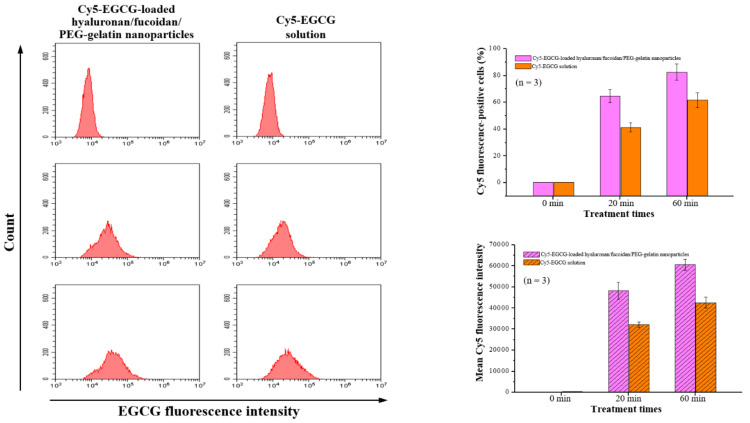
The cellular uptake and total fluorescence intensity of EGCG were examined using flow cytometry after treatment with Cy5-EGCG-loaded nanoparticles or Cy5-EGCG solution. Data are presented as means ± SDs (*n* = 3).

Flow cytometry analysis was performed with macrophages after time course treatment with Cy5-EGCG-loaded hyaluronan/fucoidan/PEG–gelatin nanoparticles. These results showed that the cellular uptake and total fluorescence intensities increased in a time-dependent manner: The cellular uptake ratio and fluorescence intensity of Cy5-EGCG were 64.6% ± 4.8% and 48,202.9 ± 3956.6, respectively, after 20 min of exposure, and these reached 82.6% ± 6.1% and 60,676.2 ± 2564.9 after the exposure time was increased to 60 min. In contrast, the mean fluorescence intensity of the Cy5-EGCG-only solution was only 32,171.3 ± 1356.6 after 20 min, reaching 42588.9 ± 2532.7 after 60 min (Figure 6). This difference between drug absorption capacities is closely related to the presence of nanoparticles containing EGCG, which successfully recognize their specific target CD44 on the cell surface.

### 2.5. Anti-Inflammatory Effects of EGCG-Loaded Nanoparticles

**Figure 7:**
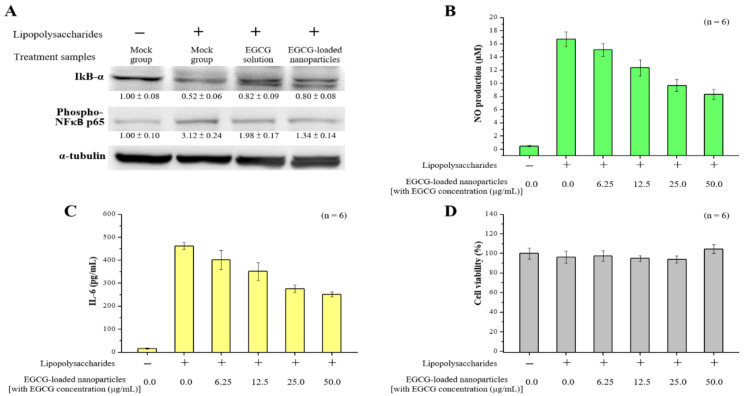
Effects of EGCG-loaded nanoparticles on lipopolysaccharide-induced pro-inflammatory mediator productions. (**A**) Western blotting analysis of inflammation-related proteins in macrophage cells after incubation with EGCG solution or EGCG-loaded nanoparticles; (**B**) NO production level; (**C**) interleukin (IL)-6 level; and (**D**) Cell viability as determined at 24 h. Data are presented as means ± SDs (*n* = 6).

The inhibitor kappa B (IκB) signaling pathway is known to play important roles in cell inflammation and proliferation. Therefore, we sought to determine whether EGCG solution or EGCG-loaded nanoparticles (with EGCG concentration 50.0 μg/mL) would inhibit the IκB signaling pathway in RAW264.7 macrophages activated by LPS. The level of IκB-α decreased in response to the presence of lipopolysaccharide in cells. Furthermore, treatment with EGCG-loaded nanoparticles significantly reduced the degradation of IκB-α compared to cells after LPS treatment (without setting the lipopolysaccharide treatment group to 100%, each independent experiment had *n* = 3, *p* < 0.05). We also examined whether the decreased degradation of IκB-α by EGCG-loaded nanoparticles might lead to decreases in nuclear factor-kappa B (NF-κB) migration into the nuclei. We found that the level of p65, one component of NF-κB, was significantly inhibited by EGCG-loaded nanoparticles, even though there was some discrepancy between IκB-α degradation and the p65 level in the lipopolysaccharide-Indo treated group (Figure 7A). Finally, we investigated the production of NO and interleukin-6 (IL-6) cytokines in lipopolysaccharide-stimulated RAW 264.7 cells. These data showed that pretreatment of EGCG-loaded nanoparticles in a concentration-dependent manner could significantly inhibit the production of pro-inflammatory NO and IL-6 in macrophages stimulated by lipopolysaccharide without affecting cell viability (Figure 7B–D). Furthermore, the pure fucoidan solution showed a low inhibitory effect on NO production. The highest dose of fucoidan treatment (50.0 μg/mL) only reduced the amount of NO produced by 10% in macrophages. This suggests that the anti-inflammatory effect of EGCG was not affected by encapsulation in nanoparticles.

## 3. Discussion

Inflammation is a response of the immune system to harmful stimuli, such as pathogens, damaged cells, toxic compounds, or irradiation, which acts to remove injurious stimuli and initiate the healing process [34,35]. The inflammatory response involves a highly coordinated network of many cell types. Among these, monocytes can differentiate into macrophages and dendritic cells, which are recruited into damaged tissues via chemotaxis. Inflammation-mediated immune cell alterations are associated with many diseases, including asthma, cancer, chronic inflammatory diseases, atherosclerosis, and autoimmune and degenerative diseases [36]. Migration of these cells from the blood to the site of inflammation is regulated by adhesion molecules. One of these is CD44, and it has been observed using murine models that the recruitment of macrophages and neutrophils into sites of inflammation is CD44-dependent [37,38,39]. Hyaluronan is a linear glycosaminoglycan that is formed by alternating disaccharide units of repeating (1→3)-β-d-GlcNAc and (1→4)-β-d-GlcA residues [40,41]. Some studies have shown that hyaluronan of different molecular weights can be covalently attached to the surface of nanoparticles to engage the activity of targeted hyaluronan receptors (CD44 and CD168), which are used in various carcinoma cells (e.g., ovarian, colon, and stomach) or innate immune cells (e.g., macrophages) [42,43,44]. Therefore, in the current study, we examined the hyaluronan binding of CD44 protein and macrophages at various hyaluronan concentrations. Our results show that the binding capacity of hyaluronan to these increases with the dosage of hyaluronan used, while the addition of the competitive CD44 antibody decreases this capacity significantly (Figure 2). Moreover, some studies have shown that hyaluronan-based nanoparticles can bind with the cell surface CD44 and are endocytosed into the endosomes and lysosomes [45,46]. It is known that hyaluronan can be digested inside the cells, presumably by acid-active lysosomal hyaluronidases [47]. Thus, the hyaluronan on nanoparticles can be partially cleaved, which will release the hyaluronan fragments and slightly adhere to the nuclei. This action is supported by the observation from Kamat et al. and Evanko et al. that hyaluronan added to the permeabilized fibroblast cells or monocytic cells can be found in the cell nuclei [45,48].

Researchers in the field of nanomedicine are intensively studying the development and design of nanoparticles that can deliver therapeutic agents to target sites [49]. Here, we used hyaluronan/fucoidan complexes with PEG–gelatin to form appropriate structures of encapsulated EGCG. The present nanoparticle system comprising hyaluronan/fucoidan/PEG–gelatin/EGCG at a ratio of 1.25:1.25:3.75:2.00 mg/mL had a suitable particle size distribution and was associated with an EGCG loading efficiency of 52.08 ± 5.37% (Table 1). Fucoidan is a water-soluble heteropolysaccharide composed of L-fucose and sulfate groups, with L-fucose-4-sulfate being the main monosaccharide component, while other monosaccharides including uronic acid, galactose, xylose, mannose, rhamnose, glucose, arabinose, and xylose are also present [50]. Fucoidan therefore possesses a range of biological activities and has been reported to be an anti-coagulant, anti-thrombotic, anti-angiogenic, anti-proliferative, and anticancer agent [51,52]. Previous work by Tengdelius et al. demonstrated that fucoidan-coated gold nanoparticles display good colloidal stability and show promising anticancer properties [53]. Fucoidan is also recognized as a ligand of macrophage scavenger receptors, so macrophages may be a primary target for its immunomodulatory effects [54]. Here, we found that fucoidan could significantly inhibit macrophage migration induced by bacterial lipopolysaccharide (Figure 1). Further, our Cy5-EGCG-loaded FLA-hyaluronan/R6G-fucoidan/VT750-PEG–gelatin nanoparticles specifically bound and interacted with macrophage cells, resulting in greater fluorescence levels of Cy5−EGCG within cells compared to in a Cy5−EGCG solution (Figure 5 and Figure 6).

To determine the mechanisms affecting macrophage inflammation resulting from our targeted EGCG-loaded nanoparticles, we measured the concentrations of inflammatory-related cytokine and proteins. Green tea (*Camellia sinensis* L.) is a popular beverage that is consumed worldwide, and its possible health benefits have been the subject of considerable attention [55,56]. Numerous benefits have been attributed to the broad biological activities of polyphenols, including but not limited to anti-inflammatory, immunomodulatory, antioxidant, cardiovascular protective, and anticancer properties [57,58]. Similarly, EGCG blocks the activation of NF-κB transcription factor (which regulates genes involved in the inflammatory response) in murine macrophages or human epithelial cells, and then downregulates the expression of inducible nitric oxide synthase (iNOS) or NO production in macrophages, resulting in immunomodulation ability [59,60]. The expression of a large number of genes involved in inflammation, such as vascular endothelial growth factor, pro-inflammatory cytokines (e.g., IL-6 and TNFα), and other agents involved in proliferation and invasion, is controlled by NF-κB [57,61,62]. Our results revealed that treatment with EGCG-loaded nanoparticles can reduce NF-κB transport, and this is related to the observed reductions in IL-6 and NO production, indicating that EGCG exerts inhibitory effects on NF-κB activation (Figure 7). Further in vivo studies will be of paramount importance to continue developing our understanding of the potential of such nanoparticles for the development of new and alternative anti-inflammatory treatments.

## 4. Materials and Methods

### 4.1. Materials

Hyaluronan (molecular weight 200 kDa) was purchased from Lifecore Biomedical, LLC (Chaska, MN, USA). Fucoidan was purchased from ChamBio (Taichung, Taiwan). Fucoidan with high molecular weight (purity ≥ 95%, molecular weights 20~200 kDa) was purified from *Fucus vesiculosus* (Sigma-Aldrich, St Louis, MO, USA). Type A gelatin (molecular weight 25 kDa), 3-(4,5-dimethyl-thiazol-yl)-2,5-diphenyltetrazolium bromide (MTT), fetal bovine serum (FBS), bovine serum albumin (BSA), phosphate-buffered saline (PBS), FLA, R6G, DAPI, and EGCG were purchased from Sigma-Aldrich (St. Louis, MO, USA). All chemicals and reagents were of analytical grade.

### 4.2. Assay of the Effects of Fucoidan on Macrophage Cell Migration

The murine macrophage cell line RAW 264.7 (ATCC^®^ TIB-71™) was purchased from the American Type Culture Collection (Manassas, VA, USA) and cells were cultured in Dulbecco’s modified Eagle’s medium (DMEM; Gibco lnc., New York, NJ, USA) supplemented with 10% FBS, 2 mM glutamine, penicillin (100 U/mL), and streptomycin (100 μg/mL) in a humidified atmosphere of 5% CO_2_ at 37 °C and then harvested for future experiments. Macrophages were resuspended in culture medium at a density of 3 × 10^5^/mL and added into the upper compartment of Transwell inserts (6 well plate, 8 µm pores; Millipore Corp., Bedford, MA, USA) for 12 h of incubation. To analyze the effects of fucoidan on cell migration, cells were incubated with different concentrations of fucoidan solution (1.0 mL, 0–100 μg/mL) for 1 h, washed with prewarmed PBS, and then added to 1.0 mL of serum-free medium with 0.2 μg/mL LPS into the upper compartment of a Transwell insert, and 1.5 mL of cultured medium containing 10% FBS was added into the lower chamber of the plate. After incubating at 37 °C in 5% CO_2_ for an additional 23 h, the migrated cells on the lower surface of the filter were fixed by 3.75% paraformaldehyde at room temperature and stained with 0.2% crystal violet for 10 min. Five fields of each well were selected at random and imaged using a light microscope (magnification, 100×), and cell numbers were counted.

### 4.3. Assay of *Hyaluronan* Binding to CD44 Protein and Macrophage Cells

To evaluate the effects of hyaluronan on the targeting selectivity of the CD44 protein, fluorescent R6G-labeled hyaluronan (R6G-hyaluronan) was synthesized in reactions between the carboxylic acid groups of hyaluronan and the amine groups of R6G. R6G (0.5 mg) was completely dissolved in 1 mL of acetonitrile, and hyaluronan (25 mg) was fully dissolved in 5 mL of distilled water. Subsequently, the R6G solutions were added to the hyaluronan solutions, and 1 mg of 1-(3-(dimethylaminopropyl)-3-ethylcarbodiimide hydrochloride was added while stirring at 4 °C. To remove unconjugated fluorescent dye, R6G-hyaluronan was dialyzed against distilled water in the dark until no fluorescence was detected in the supernatant, and the resulting R6G-hyaluronan was obtained as a powder by freeze-drying.

To assess the ability to bind to CD44, human recombinant CD44 (0.5 µg/50 µL) was added to high-hydrophobicity 96-well enzyme-linked immunosorbent plates and incubated at 4 °C overnight. The wells were first washed with PBS and incubated with 0.1 mL BSA for 60 min. The wells were then gently washed three times with PBS and incubated with different concentrations of fluorescence R6G-hyaluronan solution (10–40 µg/mL; 50 µL/well) for 30 min. The wells were gently washed three times with PBS. Then, the fluorescence intensity was measured with an EnSpire™ Multilabel Plate Reader by averaging 16 reads per well (excitation 525 nm/emission 555 nm). For the competitive binding assay, a fluorescence R6G-hyaluronan solution (40 µg/mL) and Anti-CD44 antibody (0.1 µg/mL) were added to the wells of the previously coated CD44 protein. The total volume of the reaction mixture in each well was 50 μL, and fluorescence measurements were performed after 30 min of incubation. Meanwhile, to assess the binding of hyaluronan molecules to CD44-expressing macrophage cells, RAW 264.7 cells were plated on 24 well plates for 24 h and co-treated with R6G-hyaluronan molecules or combined with Anti-CD44 antibody for 30 min of incubation. After treatment, the cells were washed three times with PBS and solubilized with 1 mL of 0.5% Triton X-100 in 0.2 M sodium hydroxide. The cell-associated test samples were quantified by analyzing cell lysates in a microplate spectrofluorometer.

### 4.4. Preparation and Characterization of Nanoparticles

To design a CD44-targeted and cell migration-inhibiting nanocarrier, we developed nanoparticles composed of fucoidan/hyaluronan complexes within PEG–gelatin of encapsulated hydrophilic EGCG. First, the PEG–gelatin was produced by adding 0.3 g of mPEG_5000_-NHS to gelatin solution in dimethyl sulfoxide (1.0 g/10.0 mL) while stirring for 4 h. The PEG–gelatin was purified by dialyzing against deionized water in the dark, with the water replaced once each day for 5 days to remove the unconjugated materials. After freeze-drying, the PEG–gelatin powder was collected and the chemical structure was identified using FTIR spectroscopy. Then, to determine the optimal preparation conditions for EGCG-loaded hyaluronan/fucoidan/PEG–gelatin nanoparticles, we investigated the incorporation of the different proportions of hyaluronan/fucoidan into the preparation of nanoparticles. The nanoparticles were produced by dropping the aqueous EGCG solution into an aqueous hyaluronan/fucoidan/PEG–gelatin mixed solution. Table 1 summarizes the different nanoparticle preparation conditions along with the measured size distributions and zeta potential values for each test sample. First, a series of hyaluronan aqueous solutions (5.00, 10.00, 15.00, or 20.00 mg/mL; 0.25 mL) and fucoidan aqueous solutions (5.00, 10.00, 15.00, or 20.00 mg/mL; 0.25 mL) were added with a pipette tip to 0.50 mL of 15.00 mg/mL PEG–gelatin aqueous solution for the formation of different aqueous hyaluronan/fucoidan/PEG–gelatin concentrations (1.25/1.25/7.50, 2.50/2.50/7.50, 3.75/3.75/7.50, and 5.00/5.00/7.50 by mg/mL, 1.00 mL). Then, aqueous EGCG (4.00 mg/mL, 1.00 mL) solution was added separately to the mixed solution with different aqueous hyaluronan/fucoidan/PEG–gelatin solutions after stirring up for 30 min at 37 °C to produce EGCG–loaded hyaluronan/fucoidan/PEG–gelatin nanoparticles at the following hyaluronan/fucoidan/PEG–gelatin/EGCG ratios (in mg/mL): 0.625:0.625:3.750:2.000, 1.250:1.250:3.750:2.000, 1.875:1.875:3.750:2.000, and 2.500:2.500:3.750:2.000. It has been reported that gelatin is a natural polymer that contains a large number of glycine residues and major amino acids such as proline and 4-hydroxyproline residues. Gelatin–polyphenol interaction occurs primarily via hydrogen bonding between hydrophobic amino acids, mostly proline and residues, and the phenol rings of polyphenols [33,63]. The nanoparticles produced were collected by centrifugation for 1 h and their particle size distributions and zeta potentials were measured with a zetasizer instrument. The amount of free EGCG in the supernatant was detected using a high-performance liquid chromatography (HPLC) system equipped with a reversed phase C18 column. Compounds were eluted with 5% acetic acid–acetonitrile (50:50, *v*/*v*) at a flow rate of 1.0 mL/min. The drug loading efficiency of the nanoparticles was calculated using the following equation: loading efficiency = (total amount of drug − amount of free drug in supernatant)/total amount of drug. The nanoparticle composition and morphology were confirmed by FTIR and transmission electron microscope (TEM) analysis.

### 4.5. Cellular Internalization of Nanoparticles and the Uptake of EGCG

The cellular distribution of fluorescence EGCG-loaded nanoparticles in macrophages was detected using fluorescence microscopy. The fluorescent polymer cyanine 5 hydrazide-labeled EGCG (Cy5-EGCG) was synthesized by adding Cy5 hydrazide in 0.5 mg/0.5 mL solution to EGCG solution (25.0 mg/5.0 mL) gradually, with continuous stirring at 4 °C in the dark for 12 h. To remove the unconjugated fluorescent dye, the Cy5–EGCG was freeze-dried, added to deionized water for the precipitation of Cy5 dye, and separated by centrifugation. The precipitation procedure was repeated three times until no fluorescence dye precipitation was found. The Cy5–EGCG solution was lyophilized with a freeze dryer. Another fluorescent dye-labeled polymer was synthesized in reactions between amine groups of fluorescent dyes (FLA or Rh6G) and carboxylic acid groups of hyaluronan and fucoidan to form FLA–hyaluronan or R6G–fucoidan. These fluorescent dyes (0.5 mg/mL) were completely dissolved in acetonitrile and added to aqueous hyaluronan or fucoidan solution (25.0 mg/5.0 mL). Then, 1-(3-(dimethylaminopropyl)-3-ethylcarbodiimide hydrochloride (1.0 mg) was added to the solutions under stirring at 4 °C. Meanwhile, the synthesis of vivotag 750-NHS ester (VT750)-PEG–gelatin was based on the reaction between *N*-hydroxysuccinimide (NHS)-ester linkage to free amines on PEG–gelatin. A solution of 0.1 mg VT750–NHS in 0.2 mL dimethyl sulfoxide was added gradually to soluble PEG–gelatin (0.2 g/20.0 mL in deionized water) with continuous stirring at 4 °C for 12 h in the dark. To remove unconjugated fluorescent dyes, these resulting products were dialyzed against deionized water until no fluorescence was detected in the supernatant and then freeze-dried to form a powder. The fluorescent Cy5-EGCG-loaded FLA-hyaluronan/R6G-fucoidan/VT750-PEG–gelatin nanoparticles were produced according to the procedure described above.

To observe the interaction of the prepared nanoparticles with RAW264.7 cells, 2 × 10^4^ cells/1.0 mL were cultured on glass coverslips at 37 °C for 24 h. Cells were treated with either the prepared fluorescent Cy5-EGCG-loaded FLA-hyaluronan/R6G-fucoidan/VT750-PEG–gelatin nanoparticles or only Cy5-EGCG solution for 2 h and then fixed in 3.7% paraformaldehyde and DAPI stained in nuclei. Stained cells were observed using fluorescence microscopy with excitation wavelengths of 340, 488, 525, 633, and 750 nm. Moreover, to determine the cellular uptake ratio, the RAW264.7 cells incubated with fluorescence Cy5-EGCG-loaded nanoparticles or Cy5-EGCG solution for either 0, 20, or 60 min were analyzed using flow cytometry. Cells were collected and washed with PBS three times. After centrifuging, cells were suspended in 0.5 mL of PBS. The Cy5-EGCG contents in 1.0 × 10^4^ cells were detected with Beckman Coulter CytoFLEX, and analysis was performed with CytExpert software.

### 4.6. Western Blotting Analysis of Inflammatory-Related Proteins

RAW264.7 cells were inoculated into 6 cm dishes at a density of 1 × 10^6^ cells per well for 24 h to achieve attached growth. Cells were then incubated with EGCG-loaded nanoparticles or only EGCG solution (with EGCG 50.0 μg/mL) for 1 h, followed by LPS (0.2 μg/mL) treatment for 23 h by cell growth. Cells were then lysed in radioimmunoprecipitation assay buffer containing phosphatase inhibitor, and a Bradford protein assay was used to quantify proteins. Equal amounts of protein were separated electrophoretically using 12% sodium dodecyl sulfate polyacrylamide gel, and then the separated proteins were transferred to a polyvinylidene difluoride membrane, which was blocked using PBS buffer containing 5% (*w*/*v*) defatted dry milk for 1 h. The blots were then probed with rabbit antibodies for IκB-α and phospho-NF-κB p65 and mouse antibody for α-tubulin (Cell Signaling Technology, Inc., Danvers, MA, USA) overnight at 4 °C while undergoing incubation. Membranes were incubated with horseradish peroxidase-conjugated secondary antibodies for 1 h, and immune complexes were detected using enhanced chemiluminescence. Then, ImageJ software was used to measure the optical density.

### 4.7. Assay of Inflammatory Cytokine *Interleukin*-*6* (*IL*-*6*) and Nitric Oxide (NO) Levels

RAW264.7 macrophages were incubated into 6 well plates at a density of 3 × 10^5^ cells per well for 24 h to achieve attached growth. Cells were then incubated with different EGCG concentrations (1.0 mL, 0~50 μg/mL) in EGCG-loaded nanoparticles or fucoidan (1.0 mL, 0~50 μg/mL) in fucoidan-only solution for 1 h and washed with prewarmed PBS, followed by LPS (0.2 μg/mL) treatment for 23 h, after which NO production was assessed by the Griess assay. NO production was estimated spectrophotometrically by measuring the accumulation of nitrites in the cultured supernatants by the Griess reaction [64]. Briefly, 100 μL of culture supernatant was mixed with 100 μL of the Griess reagent for 5 min at room temperature. The absorbance was measured at 550 nm with the *EnSpire™* Multilabel Plate Reader (PerkinElmer, Inc., Waltham, MA, USA), and the nitrite concentration was calculated using a standard curve prepared with sodium nitrite. Then, the supernatant culture was collected and quantified by using the IL-6 Mouse ELISA Kit purchased from Invitrogen (Carlsbad, CA, USA). The concentration of IL-6 was determined using a standard curve.

### 4.8. Statistical Analysis

Data are expressed as the means ± standard deviations (SD). One-way analysis of variance (ANOVA) followed by Tukey analysis was used to make pairwise comparisons between the groups. Statistical significance was set at *p* < 0.05.

## 5. Conclusions

The present study indicates that hyaluronan/fucoidan-based nanoparticles including PEG–gelatin work successfully and have the potential to act as an EGCG delivering system. Hyaluronan and fucoidan molecules have been shown to bind CD44 proteins and inhibit cell migration in macrophages. We found that the nanoparticles bind to macrophages and deliver more EGCG, confirming the potential anti-inflammatory effects of these nanoparticles in lipopolysaccharide-stimulated macrophages.

## Figures and Tables

**Table 1:** Effects of hyaluronan/fucoidan proportions on particle size, polydispersity indices, zeta potential values, and drug-loading efficiency of (−)-epigallocatechin 3-gallate (EGCG) loaded hyaluronan/fucoidan/polyethylene glycol-grafted gelatin (PEG-gelatin) nanoparticles (*n* = 5).

PEG–Gelatin 3.750 mg/mL; EGCG 2.000 mg/mL				
Hyaluronan/Fucoidan (mg/mL)	Mean Particle Size (nm)	Polydispersity Indices	Zeta Potential Value (mV)	Egcg Loading Efficiency (%)
0.625:0.625	244.00 ± 12.00	0.30 ± 0.05	−29.86 ± 1.56	39.86 ± 4.88
1.250:1.250	217.00 ± 14.00	0.28 ± 0.07	−33.60 ± 1.30	52.08 ± 5.37
1.875:1.875	279.00 ± 16.00	0.38 ± 0.02	−37.76 ± 0.67	62.66 ± 3.21
2.500:2.500	454.00 ± 28.00	0.45 ± 0.10	−37.72 ± 1.16	70.12 ± 2.09

## Abbreviations

BSA	bovine serum albumin
Cy5	cyanine 5 hydrazide
DAPI	4′,6*-*diamidino*-*2*-*phenylindole
EGCG	(−)-epigallocatechin 3-gallate
FLA	fluoresceinamine
FBS	fetal bovine serum
FTIR	Fourier transform infrared spectroscopy
HPLC	high-performance liquid chromatography
IL-6	interleukin*-*6
LPS	lipopolysaccharides
NO	nitric oxide
NF-κB	nuclear factor-kappa B
PEG	polyethylene glycol
PBS	phosphate-buffered saline
R6G	rhodamine 6G
SD	standard deviation
TEM	transmission electron microscope
VT750	vivotag *750-*N*HS* ester

## References

[1] Caprara G., Allavena P., Erreni M. (2020). Intestinal Macrophages at the Crossroad between Diet, Inflammation, and Cancer. Int. J. Mol. Sci..

[2] Lavin Y., Mortha A., Rahman A., Merad M. (2015). Regulation of Macrophage Development and Function in Peripheral Tissues. Nat. Rev. Immunol..

[3] Gregory C., Devitt A. (2004). The Macrophage and the Apoptotic Cell: An Innate Immune Interaction Viewed Simplistically?. Immunology.

[4] Sica A., Mantovani A. (2012). Macrophage Plasticity and Polarization: In vivo Veritas. J. Clin. Investig..

[5] Shields C.W., Wang L.L.W., Evans M.A., Mitragotri S. (2020). Materials for Immunotherapy. Adv. Mater..

[6] He W., Kapate N., Shields C.W., Mitragotri S. (2019). Drug Delivery to Macrophages: A Review of Targeting Drugs and Drug Carriers to Macrophages for Inflammatory Diseases. Adv. Drug Deliv. Rev..

[7] Antonov A.S., Kolodgie F.D., Munn D.H., Gerrity R.G. (2004). Regulation of Macrophage Foam Cell Formation by alphaVbeta3 Integrin: Potential Role in Human Atherosclerosis. Am. J. Pathol..

[8] Akira S., Uematsu S., Takeuchi O. (2006). Pathogen Recognition and Innate Immunity. Cell.

[9] Gee K., Lim W., Ma W., Nandan D., Diaz-Mitoma F., Kozlowski M., Kumar A. (2002). Differential Regulation of CD44 Expression by Lipopolysaccharide (LPS) and TNF-α in Human Monocytic Cells: Distinct Involvement of c-Jun N-Terminal Kinase in LPS-induced CD44 Expression. J. Immunol..

[10] Naor D., Sionov R.V., Ish-Shalom D. (1997). CD44: Structure, Function, and Association with the Malignant Process. Adv. Cancer Res..

[11] Puré E., Cuff C.A. (2001). A Crucial Role for CD44 in Inflammation. Trends Mol. Med..

[12] Siegelman M., DeGrendele H., Estess P. (1999). Activation and Interaction of CD44 and Hyaluronan in Immunological Systems. J. Leukoc. Biol..

[13] Cunha L., Grenha A. (2016). Sulfated Seaweed Polysaccharides as Multifunctional Materials in Drug Delivery Applications. Mar. Drugs.

[14] Zayed A., Ulber R. (2020). Fucoidans: Downstream Processes and Recent Applications. Mar. Drugs.

[15] Sakai T., Ishizuka K., Shimanaka K., Ikai K., Kato I. (2003). Structures of oligosaccharides derived from Cladosiphon Okamuranus Fucoidan by Digestion with Marine Bacterial Enzymes. Mar. Biotechnol..

[16] Zhao Y., Zheng Y., Wang J., Ma S., Yu Y., White W.L., Yang S., Yang F., Lu J. (2018). Fucoidan Extracted from *Undaria pinnatifida*: Source for Nutraceuticals/Functional Foods. Mar. Drugs.

[17] Kim K.J., Lee B.Y. (2012). Fucoidan from the Sporophyll of Undaria Pinnatifida Suppresses Adipocyte Differentiation by Inhibition of Inflammation-Related Cytokines in 3T3-L1 Cells. Nutr. Res..

[18] Synytsya A., Bleha R., Synytsya A., Pohl R., Hayashi K., Yoshinaga K., Nakano T., Hayashi T. (2014). Mekabu Fucoidan: Structural Complexity and Defensive Effects Against Avian Influenza A viruses. Carbohydr. Polym..

[19] Lu M.K., Cheng J.J., Lin C.Y., Chang C.C. (2010). Purification, Structural Elucidation, and Anti-Inflammatory Effect of a Water-Soluble 1,6-Branched 1,3-α-*d*-Galactan from Cultured Mycelia of Poria Cocos. Food Chem..

[20] Xue M., Ge Y., Zhang J., Liu Y., Wang Q., Hou L., Zheng Z. (2013). Fucoidan Inhibited 4T1 Mouse Breast Cancer Cell Growth in Vivo and in Vitro via Downregulation of Wnt/β-catenin signaling. Nutr. Cancer.

[21] Park J.H., Choi S.H., Park S.J., Lee Y.J., Park J.H., Song P.H., Cho C.M., Ku S.K., Song C.H. (2017). Promoting Wound Healing Using Low Molecular Weight Fucoidan in a Full-Thickness Dermal Excision Rat Model. Mar. Drugs.

[22] Li P., Wang H., Shao Q., Kong B., Qu X. (2017). Fucoidan Modulates Cytokine Production and Migration of THP-1-Derived Macrophages via Colony-Stimulating Factor-1. Mol. Med. Rep..

[23] Zhang X.Q., Kim J.H., Lee G.S., Pyo H.B., Shin E.Y., Kim E.G., Zhang Y.H. (2012). In Vitro Antioxidant and In Vivo Anti-Inflammatory Activities of *Ophioglossum thermale*. Am. J. Chin. Med..

[24] Exarchou V., Nenadis N., Tsimidou M., Gerothanassis I.P., Troganis A., Boskou D. (2002). Antioxidant Activities and Phenolic Composition of Extracts from Greek Oregano, Greek Sage, and Summer Savory. J. Agric. Food Chem..

[25] Muzolf M., Szymusiak H., Gliszczyńska-Swigło A., Rietjens I.M., Tyrakowska B. (2008). pH-Dependent Radical Scavenging Capacity of Green Tea Catechins. J. Agric. Food Chem..

[26] Tang D.W., Yu S.H., Ho Y.C., Huang B.Q., Tsai G.J., Hsieh H.Y., Sung H.W., Mi F.L. (2013). Characterization of Tea Catechins-Loaded Nanoparticles Prepared from Chitosan and an Edible Polypeptide. Food Hydrocoll..

[27] Ananingsih V., Sharma A., Zhou W. (2013). Green Tea Catechins during Food Processing and Storage: A Review on Stability and Detection. Food Res. Int..

[28] Zhong Y., Chiou Y.S., Pan M.H., Shahidi F. (2012). Anti-Inflammatory Activity of Lipophilic Epigallocatechin Gallate (EGCG) Derivatives in LPS-stimulated Murine Macrophages. Food Chem..

[29] Syed D.N., Afaq F., Kweon M.H., Hadi N., Bhatia N., Spiegelman V.S., Mukhtar H. (2007). Green Tea Polyphenol EGCG Suppresses Cigarette Smoke Condensate-Induced NF-kappaB Activation in Normal Human Bronchial Epithelial Cells. Oncogene.

[30] Chen C.C., Hsieh D.S., Huang K.J., Chan Y.L., Hong P.D., Yeh M.K., Wu C.J. (2014). Improving Anticancer Efficacy of (-)-Epigallocatechin-3-Gallate Gold Nanoparticles in Murine B16F10 Melanoma Cells. Drug Des. Dev. Ther..

[31] Landis-Piwowar K., Huo C., Chen D., Milacic V., Shi G., Chan T.H., Dou Q.P. (2007). A Novel Prodrug of the Green Tea Polyphenol (-)-Epigallocatechin-3-Gallate as a Potential Anticancer Agent. Cancer Res..

[32] Huang W.Y., Lin J.N., Hsieh J.T., Chou S.C., Lai C.H., Yun E.J., Lo U.G., Pong R.C., Lin J.H., Lin Y.H. (2016). Nanoparticle Targeting CD44-Positive Cancer Cells for Site-Specific Drug Delivery in Prostate Cancer Therapy. ACS Appl. Mater. Interfaces.

[33] Shutava T.G., Balkundi S.S., Vangala P., Steffan J., Bigelow R.L., Cardelli J.A., O’Neal D.P., Lvov Y.M. (2009). Layer-by-Layer-Coated Gelatin Nanoparticles as a Vehicle for Delivery of Natural Polyphenols. ACS Nano.

[34] Medzhitov R. (2010). Inflammation 2010: New Adventures of an Old Flame. Cell.

[35] Ferrero-Miliani L., Nielsen O.H., Andersen P.S., Girardin S.E. (2007). Chronic Inflammation: Importance of NOD2 and NALP3 in Interleukin-1β Generation. Clin. Exp. Immunol..

[36] Chen L., Deng H., Cui H., Fang J., Zuo Z., Deng J., Li Y., Wang X., Zhao L. (2018). Inflammatory Responses and Inflammation-Associated Diseases in Organs. Oncotarget.

[37] Sladek Z., Rysanek D. (2009). Expression of Macrophage CD44 Receptor in the Course of Experimental Inflammatory Response of Bovine Mammary Gland Induced by Lipopolysaccharide and Muramyl Dipeptide. Res. Vet. Sci..

[38] Alstergren P., Zhu B., Glogauer M., Mak T.W., Ellen R.P., Sodek J. (2004). Polarization and Directed Migration of Murine Neutrophils is Dependent on Cell Surface Expression of CD44. Cell Immunol..

[39] Hollingsworth J.W., Li Z., Brass D.M., Garantziotis S., Timberlake S.H., Kim A., Hossain I., Savani R.C., Schwartz D.A. (2007). CD44 Regulates Macrophage Recruitment to the Lung in Lipopolysaccharide-Induced Airway Disease. Am. J. Respir. Cell Mol. Biol..

[40] Nagarajan B., Sankaranarayanan N.V., Desai U.R. (2019). Perspective on Computational Simulations of Glycosaminoglycans. Wiley Interdiscip. Rev. Comput. Mol. Sci..

[41] Harris E.N., Baker E. (2020). Role of the Hyaluronan Receptor, Stabilin-2/HARE, in Health and Disease. Int. J. Mol. Sci..

[42] Platt V.M., Szoka F.C. (2008). Anticancer Therapeutics: Targeting Macromolecules and Nanocarriers to Hyaluronan or CD44, a Hyaluronan Receptor. Mol. Pharm..

[43] Shigeishi H., Fujimoto S., Hiraoka M., Ono S., Taki M., Ohta K., Higashikawa K., Kamata N. (2009). Overexpression of the Receptor for Hyaluronan-Mediated Motility, Correlates with Expression of Microtubule-Associated Protein in Human Oral Squamous Cell Carcinomas. Int. J. Oncol..

[44] Mizrahy S., Raz S.R., Hasgaard M., Liu H., Soffer-Tsur N., Cohen K., Dvash R., Landsman-Milo D., Bremer M.G.E.G., Moghimi S.M. (2011). Hyaluronan-Coated Nanoparticles: The Influence of the Molecular Weight on CD44-Hyaluronan Interactions and on the Immune Response. J. Control. Release.

[45] Kamat M., El-Boubbou K., Zhu D.C., Lansdell T., Lu X., Li W., Huang X. (2010). Hyaluronic Acid Immobilized Magnetic Nanoparticles for Active Targeting and Imaging of Macrophages. Bioconj. Chem..

[46] Montanari E., Mancini P., Galli F., Varani M., Santino I., Coviello T., Mosca L., Matricardi P., Rancan F., Meo C.D. (2020). Biodistribution and Intracellular Localization of Hyaluronan and its Nanogels. A Strategy to Target Intracellular, S. Aureus in Persistent Skin Infections. J. Control. Release.

[47] Lepperdinger G., Strobl B., Kreil G. (1998). HYAL2, A Human Gene Expressed in Many Cells, Encodes a Lysosomal Hyaluronidase with a Novel Type of Specificity. J. Biol. Chem..

[48] Evanko S.P., Wight T.N. (1999). Intracellular Localization of Hyaluronan in Proliferating Cells. J. Histochem. Cytochem..

[49] Patra J.K., Das G., Fraceto L.F., Campos E.V.R., Rodriguez-Torres M.D.P., Acosta-Torres L.S., Diaz-Torres L.A., Grillo R., Swamy M.K., Sharma S. (2018). Nano Based Drug Delivery Systems: Recent Developments and Future Prospects. J. Nanobiotechnol..

[50] Wang Y., Xing M., Cao Q., Ji A., Liang H., Song S. (2019). Biological Activities of Fucoidan and the Factors Mediating Its Therapeutic Effects: A Review of Recent Studies. Mar. Drugs.

[51] Kwak J.Y. (2014). Fucoidan as a Marine Anticancer Agent in Preclinical Development. Mar. Drugs.

[52] Wijesinghea W.A.J.P., Jeon Y.J. (2012). Biological Activities and Potential Industrial Applications of Fucose Rich Sulfated Polysaccharides and Fucoidans Isolated from Brown Seaweeds: A Review. Carbohydr. Polym..

[53] Tengdelius M., Gurav D., Konradsson P., Påhlsson P., Griffith M., Oommen O.P. (2015). Synthesis and Anticancer Properties of Fucoidan-Mimetic Glycopolymer Coated Gold Nanoparticles. Chem. Commun..

[54] Hsu H.Y., Chiu S.L., Wen M.H., Chen K.Y., Hua K.F. (2001). Ligands of Macrophage Scavenger Receptor Induce Cytokine Expression via Differential Modulation of Protein Kinase Signaling Pathways. J. Biol. Chem..

[55] Yang C.S., Chung J.Y., Yang G., Chhabra S.K., Lee M.J. (2000). Tea and Tea Polyphenols in Cancer Prevention. J. Nutr..

[56] Lin J.K., Liang Y.C., Lin-Shiau S.Y. (1999). Cancer Chemoprevention by Tea Polyphenols through Mitotic Signal Transduction Blockade. Biochem. Pharmacol..

[57] Yahfoufi N., Alsadi N., Jambi M., Matar C. (2018). The Immunomodulatory and Anti-inflammatory Role of Polyphenols. Nutrients.

[58] Eberhardt M.V., Lee C.Y., Liu R.H. (2000). Antioxidant Activity of Fresh Apples. Nature.

[59] Kanwar J., Taskeen M., Mohammad I., Huo C., Chan T.H., Dou Q.P. (2012). Recent Advances on Tea Polyphenols. Front. Biosci..

[60] Singh B.N., Shankar S., Srivastava R.K. (2011). Green Tea Catechin, Epigallocatechin-3-Gallate (EGCG): Mechanisms, Perspectives and Clinical. Biochem. Pharmacol..

[61] Peairs A., Dai R., Gan L., Shimp S., Rylander M.N., Li L., Reilly C.M. (2010). Epigallocatechin-3-Gallate (EGCG) Attenuates Inflammation in MRL/lpr Mouse Mesangial Cells. Cell Mol. Immunol..

[62] Nam N.H. (2006). Naturally Occurring NF-κB Inhibitors. Mini-Rev. Med. Chem..

[63] Samp M.A., Iovanac N.C., Nolte A.J. (2017). Sodium Alginate Toughening of Gelatin Hydrogels. ACS Biomater. Sci. Eng..

[64] Amano F., Noda T. (1995). Improved Detection of Nitric Oxide Radical (NO.) Production in an Activated Macrophage Culture with a Radical Scavenger, Carboxy PTIO, and Griess Reagent. FEBS Lett..

